# Correlation analysis of serum galectin-3 combined with Th1/Th2-related cytokines and severe anxiety and depression symptoms in patients with chronic obstructive pulmonary disease

**DOI:** 10.3389/fpsyt.2026.1816398

**Published:** 2026-07-24

**Authors:** Zhaodi Qiu, Bifang Huang, Hua Zhou, Yinan Huang, Huiyu Liu

**Affiliations:** 1 Department of Psychiatry, Ward II (Comorbid Medical Conditions), The Third People's Hospital of Ganzhou City, Ganzhou, Jiangxi, China; 2 Department of Respiratory and Critical Care Medicine, Ganzhou Hospital-Nanfang Hospital, Southern Medical University, Ganzhou People's Hospital, Ganzhou City, Ganzhou, Jiangxi, China

**Keywords:** anxiety, biomarkers, chronic obstructive pulmonary disease, depression, galectin-3, Th1/Th2 cytokines

## Abstract

**Objective:**

To evaluate the synergistic effect of serum Galectin-3 (Gal-3) and Th1/Th2 level-related cytokines, and its association with severe anxiety and depression symptoms in patients with chronic obstructive pulmonary disease (COPD), aiming to provide potential indicators for clinical stratified assessment.

**Methods:**

This study was a cross-sectional study. A total of 115 patients with COPD who were admitted to our hospital from August 2022 to October 2025 were selected as the research subjects. Based on the Hospital Anxiety and Depression Scale (HADS), the patients were divided into the asymptomatic group (HADS anxiety score ≤ 7 points), the mild group (8–10 points), and the moderate-to-severe group (> 10 points). Serum levels of Gal-3, Th1 cytokines (IL-2 and IFN-γ), and Th2 cytokines (IL-4 and IL-33) were quantified. Pearson correlation analysis was used to examine the relationship. Logistic regression model was employed to analyze the influencing factors. Receiver operating characteristic curve (ROC) was used to evaluate the diagnostic value of serum Gal-3 and Th1/Th2.

**Results:**

Serum levels of Gal-3, IL-2, IFN-γ, IL-4, and IL-33 were increased significantly in correlation with symptom severity, with the highest levels observed in the moderate-to-severe group (*P* < 0.05). All these markers were positively correlated with the HADS anxiety dimension scores and the HADS depression dimension scores (*P* < 0.05). Serum Gal-3, IL-2, IFN-γ, IL-4, and IL-33 were all associated factors for anxiety and depression symptoms in COPD patients (*P* < 0.05). The ROC curve revealed that the AUCs of serum indicators Gal-3, IL-2, IFN-γ, IL-4, and IL-33 for diagnosing moderate-to-severe anxiety and depression symptoms in COPD patients were 0.814, 0.767, 0.778, 0.847, and 0.823, respectively. The combined detection model achieved a superior AUC of 0.905 (95% CI: 0.845–0.965) for identifying moderate-to-severe anxiety and depression symptoms. This combined model demonstrated a sensitivity of 85.51% and a specificity of 86.96%, significantly outperforming the single-marker.

**Conclusion:**

Gal-3 was synergistic with Th1/Th2-related cytokines and was associated with the severity of anxiety and depression symptoms in patients with COPD. The combined detection of these markers may provide a reference for early clinical stratification assessment.

## 1 Introduction

Chronic obstructive pulmonary disease (COPD) is a prevalent respiratory disorder affecting approximately 10% of the global population. By 2025, the prevalence of COPD among individuals aged 25 and older is projected to rise to approximately 23% (1, 2). Anxiety and depression are common comorbidities in patients with COPD and are associated with an increased risk of acute exacerbations. Research has identified anxiety and depression as independent risk factors for these exacerbations, contributing to prolonged hospital stays and adverse prognostic outcomes (3).

Galectin-3 (Gal-3), a β-galactoside-binding lectin, plays a pivotal role in inflammation, fibrosis, and immune regulation. Evidence indicates that Gal-3 expression is upregulated in both the serum and lung tissue of patients with COPD. These elevated levels are closely correlated with the severity of airflow limitation, the frequency of acute exacerbations, and disease progression, suggesting that Gal-3 is a critical mediator in chronic airway inflammation and remodeling (4). Furthermore, Gal-3 possesses the ability to cross the blood-brain barrier, activate central microglia, and promote neuroinflammation a core mechanism underlying psychiatric disorders such as depression and anxiety (5). This suggests that Gal-3 may serve as a potential molecular bridge linking peripheral inflammation in COPD to central nervous system dysfunction.

The equilibrium between T helper 1 (Th1) and T helper 2 (Th2) cells is fundamental to the maintenance of immune homeostasis. A hallmark of COPD is a Th1/Th2 immune imbalance, characterized primarily by the excessive activation of Th1-type immune responses. Th1 dominance typically manifests as the upregulation of Th1 cells and associated cytokines, which subsequently activate macrophages, recruit neutrophils, and promote the release of proinflammatory mediators such as tumor necrosis factor-α (TNF-α). This cascade drives persistent chronic airway inflammation, tissue destruction, and airway remodeling, thereby acting as a key driver of inflammatory progression in COPD (6). While Th2 immune responses are relatively weaker in classic COPD, they are not entirely absent. Studies indicate that Th2 cytokines, such as interleukin-4 (IL-4), are elevated in a subset of COPD patients and may contribute to disease heterogeneity by promoting mucus hypersecretion and eosinophilic inflammation (7). In this study, IL-2, IFN-γ, IL-4 and IL-33 were selected from numerous Th1/Th2 cytokines as detection indicators based on the following: IL-2 is a key factor that drives the proliferation and differentiation of T cells, and it can reflect the activation intensity of Th1 immune response (8). Moreover, it is significantly correlated with depressive symptoms in patients with COPD (8). IFN-γ, as the characteristic effector molecule of Th1-type immunity, shows a dominant expression in the pulmonary inflammation of COPD and participates in neuronal apoptosis (9). IL-4 is a key upstream factor that initiates Th2 differentiation and is regarded as a sensitive indicator of Th2 response deviation (7). Although IL-33 is not a traditional Th2 differentiation factor, as an “alarm signal” released by airway epithelium, it can strongly promote Th2-type immune responses and airway inflammation (10). The above four factors collectively cover the classic Th1/Th2 pathway and the epithelial-immune interconnection pathway, and can comprehensively reflect the Th1/Th2 immune imbalance state in COPD and its potential neuro-immune effects.

Currently, it is believed that the mechanism underlying the high incidence of anxiety and depression in patients with COPD is not only related to psychological stress, but also involves a specific biological basis. Among them, the “lung-brain axis” theory is the key physiological basis connecting pulmonary pathology and central nervous system dysfunction (11). When COPD causes chronic inflammation and hypoxia in the periphery, inflammatory mediators can directly invade the brain parenchyma through the damaged blood-brain barrier, or transmit peripheral immune signals via the vagus nerve input pathway to the solitary nucleus and the locus coeruleus in the brainstem, thereby overactivating the hypothalamic-pituitary-adrenal (HPA) axis and disrupting the metabolic balance of monoamine neurotransmitters (such as serotonin and norepinephrine) (12). Furthermore, chronic hypoxemia and oxidative stress can further damage the tight junctions of cerebral vascular endothelial cells, increase the permeability of the blood-brain barrier, promote the infiltration of peripheral pro-inflammatory factors (such as IL-1β, TNF-α) into the central nervous system, and abnormally activate microglia, thereby triggering persistent neuroinflammation in the hippocampus and prefrontal cortex (13). These multiple signaling pathways collectively form the core pathological framework of the “lung-brain axis”. Therefore, investigating the influence of Gal-3 and Th1/Th2 imbalance on the anxiety and depression symptoms of COPD patients in the lung-brain axis pathway holds significant theoretical and clinical value, as it helps to elucidate how peripheral immune inflammation drives central emotional disorders through this pathway.

Although previous studies have independently examined the relationships between Gal-3 or Th1/Th2 cytokines and either anxiety/depression or COPD, there is a paucity of research integrating these factors within the specific context of chronic inflammation in COPD to jointly explore their correlation with the severity of psychiatric symptoms. Furthermore, existing literature has predominantly focused on single-marker correlation analyses, often neglecting the diagnostic value of multifactorial combinations. This limitation hinders the clinical requirement for precise identification of emotional disorders.

Therefore, this study is the first to integrate Gal-3 with Th1/Th2-related cytokines (IL-2, IFN-γ, IL-4, and IL-33). Through correlation analysis, logistic regression, and receiver operating characteristic (ROC) curve analysis, we aim to elucidate the association of these markers with the severity of anxiety and depression (including comorbidities) in COPD patients and to evaluate their combined diagnostic efficacy, thereby addressing current gaps in the literature.

## 2 Materials and methods

### 2.1 Baseline data

Sample size estimation: This study was a cross-sectional study. The main analysis objectives were: (1) Logistic regression analysis to identify the factors associated with anxiety and depression in patients with COPD; (2) ROC curve to evaluate the identification efficacy of serum indicators for moderate to severe anxiety and depression symptoms. Following the rule of at least 10 to 15 outcome events for each independent variable in Logistic regression analysis, this study initially included 7 to 9 independent variables, with the requirement that the moderate to severe anxiety and depression group should have at least 70 to 105 cases. However, according to the literature reports and the preliminary investigation conducted by our hospital, the incidence of moderate to severe anxiety and depression symptoms among patients with stable COPD was approximately 10% to 15%. Based on this proportion, the total sample size required was approximately 700 to 1000 cases, which exceeded the feasibility range of a single-center retrospective study. Therefore, this study employed a sample size estimation method based on ROC analysis: With the expected AUC of the combined test being 0.85 (based on the results of the pre-experiment), the null hypothesis AUC being 0.70, the significance level α being 0.05 (two-sided), the confidence level (1 - β) being 0.80, and the sample size ratio between the case group (moderate-to-severe group) and the control group (asymptomatic group + mild group) being 1:5, using the PASS 15.0 software for calculation, it was determined that the minimum number of cases required in the moderate-severe group was 13, and the minimum number of cases required in the control group was 65, resulting in a total sample size of at least 78 cases. This study actually included 14 cases from the moderate-to-severe group and 101 cases from the control group (46 cases from the asymptomatic group + 55 cases from the mild group), meeting the minimum sample size requirement.

**Figure 1:**
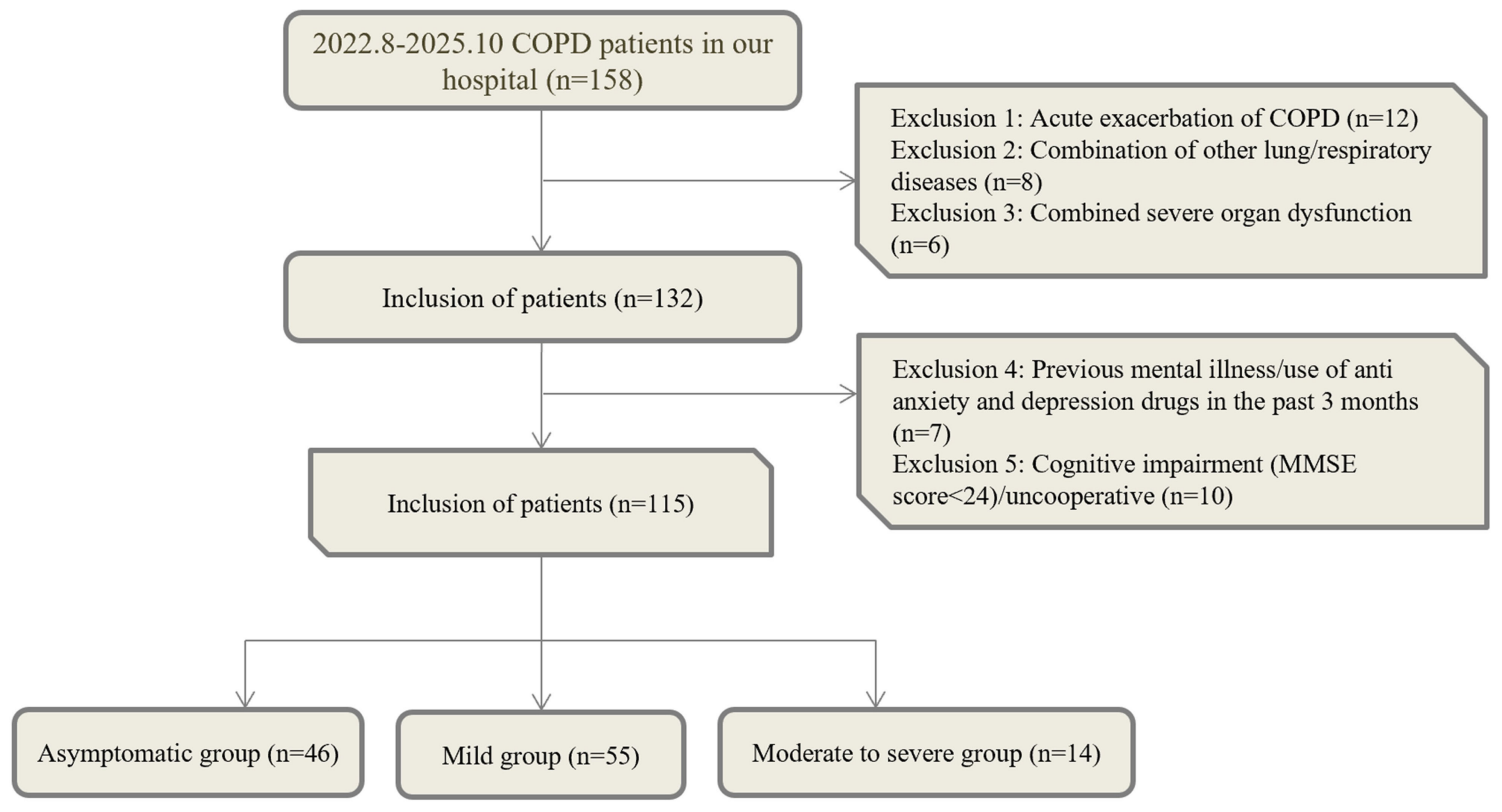
Flowchart of patient selection and enrollment.

This study was a cross-sectional study. A total of 115 patients diagnosed with COPD who were admitted to our hospital from August 2022 to October 2025 were selected as the research subjects. The patient inclusion and exclusion process was illustrated in **Figure 1**. Inclusion criteria: (1) Patients met the COPD diagnostic criteria specified in the “Chronic Obstructive Pulmonary Disease Diagnosis and Treatment Guidelines (Revised Edition 2021)” by the Chronic Obstructive Pulmonary Disease Group of the Chinese Medical Association’s Respiratory Diseases Society (14), defined as a post-bronchodilator forced expiratory volume in 1 second (FEV1) to forced vital capacity (FVC) ratio (FEV1/FVC) of <0.70 and an FEV1 <80% of the predicted value; (2) A clinical status of disease stability, characterized by no significant exacerbation of symptoms (cough, sputum production, dyspnea), no hospitalizations for acute exacerbations, and no modifications to the treatment regimen within the preceding two months; (3) Normal cognitive function, evidenced by a Mini-Mental State Examination (MMSE) score of ≥24, ensuring the ability to communicate effectively and complete the required questionnaires; (4) An age ranging from 40 to 80 years. Exclusion criteria: (1) Patients were presented with concurrent pulmonary comorbidities such as tuberculosis, pulmonary fibrosis, or lung malignancies; (2) Other respiratory disorders affecting lung function, including bronchial asthma, bronchiectasis, or pulmonary thromboembolism; (3) Severe dysfunction of major organs, such as chronic heart failure, liver cirrhosis, or chronic renal failure; an active acute exacerbation of COPD; (4) In the acute exacerbation stage of COPD; (5) A history of diagnosed psychiatric disorders (e.g., anxiety, depression) or the use of anxiolytics or antidepressants within the past three months; (6) Concurrent malignant tumors, autoimmune diseases, or acute infections within the month prior to enrollment. The study protocol was approved by the hospital ethics committee.

### 2.2 Grouping criteria

The emotional status of patients was assessed within 24 to 48 hours of admission using the Hospital Anxiety and Depression Scale (HADS) (15). This validated tool comprises two subscales: the Anxiety Scale (HAS) and the Depression Scale (HDS). Each subscale contains seven items scored from 0 to 3 based on symptom severity, yielding a total score of 0 to 21 for each dimension. In this study, the HADS was used to assess the anxiety and depression symptoms of patients within 24 to 48 hours after admission. The main basis for this was as follows: (1) Previous systematic reviews confirmed that the HADS anxiety subscale had good reliability and validity in patients with stable COPD (15). (2) The systematic review by Larsen et al. (16) demonstrated that the HADS-D had a 100% sensitivity and over 85% specificity in screening depression among COPD patients. (3) The items of the HADS scale did not include physical symptoms (such as insomnia, loss of appetite, etc.), which can effectively avoid the interference of COPD’s own physical symptoms on the assessment of emotions, thus being more suitable for screening emotional disorders in patients with physical diseases (17).

Based on their HADS, patients were stratified into three groups: the asymptomatic group (n=46), defined by HAS and HDS scores both ≤7, indicating the absence of significant anxiety or depression symptoms; the mild group (n=55), defined by HAS or HDS scores between 8 and 10, indicating the presence of mild anxiety and/or depression symptoms; and the moderate-to-severe group (n=14), defined by HAS or HDS scores >10, indicating the presence of moderate to severe anxiety and/or depressive symptoms.

### 2.3 Clinical data collection

Clinical data were extracted from the hospital electronic medical record system and structured questionnaires. The specific contents were as follows: (1) Demographic variables included age and gender; (2) Anthropometric measurements taken at admission were used to calculate Body Mass Index (BMI), which was classified according to WHO standards as underweight (≤18.4 kg/m²), normal weight (18.5–24.9 kg/m²), or overweight/obese (≥25 kg/m²); (3) Smoking history was categorized as either current/former smokers (cumulative consumption ≥100 cigarettes or cessation <1 year) or non-smokers; (4) Disease duration was stratified into <10 years and ≥10 years; (5) Respiratory function and symptom severity were evaluated using the modified Medical Research Council (mMRC) Dyspnea Scale, graded from 0 to 4. Grade 0 indicated dyspnea only with strenuous exercise, Grade 1 indicated dyspnea when hurrying on level ground or walking up a slight hill, Grade 2 indicated walking slower than people of the same age or stopping for breath when walking at own pace, Grade 3 indicated stopping for breath after walking approximately 100 meters or after a few minutes, and Grade 4 indicated being too breathless to leave the house or breathless when dressing/undressing; (6) Physical activity levels were assessed via questionnaire, with regular physical activity defined as ≥150 minutes of moderate-intensity activity (e.g., brisk walking, Tai Chi) or ≥75 minutes of vigorous-intensity activity (e.g., running) per week; (7) Comorbidities were recorded, including hypertension (systolic BP ≥140 mmHg and/or diastolic BP ≥90 mmHg, or medication use), type 2 diabetes (fasting glucose ≥7.0 mmol/L, 2-hour postprandial glucose ≥11.1 mmol/L, or medication use), and coronary heart disease (confirmed by angiography/CTA or history of myocardial infarction).

### 2.4 Measurement of serum Gal-3 and Th1/Th2 cytokines

Fasting venous blood samples (5 mL) were collected from the antecubital vein on the morning following admission. Samples were collected in anticoagulant-free vacuum tubes, allowed to clot naturally at room temperature for 30 minutes, and centrifuged at 4500 rpm for 15 minutes using a JIDI-17RS centrifuge (radius 10 cm). The serum supernatant was carefully aliquoted into enzyme-free EP tubes and stored at -80 °C to prevent repeated freeze-thaw cycles.

Serum concentrations were quantified using enzyme-linked immunosorbent assay (ELISA). The Gal-3 assay kit was obtained from Shanghai Enzyme-Linked Bio-Technology Co., Ltd. (detection range: 0.1–100 ng/mL; limit of detection [LOD]: 0.05 ng/mL). Th1 cytokines (IL-2, IFN-γ) and Th2 cytokines (IL-4, IL-33) were measured using kits from Beijing Beyotime Biotechnology Co., Ltd. The detection ranges were as follows: IL-2, 10–1000 pg/L; IFN-γ, 50–5000 ng/L; IL-4, 10–1000 ng/L; and IL-33, 50–5000 pg/mL. The LOD for all cytokines was <5% of the lowest standard concentration.

Assays were performed strictly according to manufacturer instructions. Frozen serum was thawed at 4 °C and equilibrated to room temperature for 20 minutes before dilution. Standards, samples, and antibody working solutions were added sequentially and incubated at 37 °C for 60 minutes. After five washes, the enzyme-conjugated antibody was added and incubated at 37 °C for 30 minutes. Following a second wash series, the substrate solution was added for a 15-minute incubation in the dark. The reaction was terminated with a stop solution, and optical density (OD) was measured at 450 nm using a Thermo Multiskan FC microplate reader. Concentrations were calculated from standard curves. All samples were analyzed in duplicate with blank, negative, and positive controls to ensure a coefficient of variation (CV) <10%.

### 2.5 Statistical analysis

Data analyses were performed using SPSS software (version 26.0). Continuous variables adhering to a normal distribution were presented as mean ± standard deviation (*x* ± *s*). Comparisons between two groups were conducted using the independent samples t-test, while comparisons among multiple groups utilized one-way analysis of variance (ANOVA) followed by the Least Significant Difference (LSD) *post hoc* test. Categorical variables were expressed as frequencies (percentages) and were compared using the Chi-square (χ²) test. Pearson correlation analysis was employed to assess associations between serum biomarkers (Gal-3, IL-2, IFN-γ, IL-4, IL-33) and HADS scores. A multivariate Logistic regression analysis was employed to explore the influencing factors of anxiety and depression symptoms in patients with COPD. The model adopted the “entering method” (Enter). The diagnostic value of serum markers for moderate-to-severe anxiety and depression was evaluated using Receiver Operating Characteristic (ROC) curve analysis. A two-tailed *P*-value <0.05 was considered statistically significant.

## 3 Results

### 3.1 Clinical characteristics of COPD patients stratified by anxiety and depression severity

Demographic and clinical parameters, including age, gender, BMI, smoking status, physical activity, and the presence of comorbidities, were comparable across the asymptomatic, mild, and moderate-to-severe groups, with no statistically significant differences observed (*P*>0.05). However, significant disparities were noted regarding the severity of dyspnea (mMRC grade) and the distribution of disease duration (*P*<0.05). Pairwise comparisons demonstrated that patients in both the mild and moderate-to-severe groups exhibited significantly higher mMRC grades compared to the asymptomatic group (*P*<0.05). Furthermore, mMRC grades in the moderate-to-severe group were significantly elevated compared to the mild group (*P*<0.05). Regarding disease duration, the proportion of patients with a disease history of <10 years was significantly higher in both the mild and moderate-to-severe groups compared to the asymptomatic group (*P*<0.05). Additionally, the moderate-to-severe group showed a significantly higher proportion of patients with a duration of <10 years compared to the mild group (*P* < 0.05). Detailed data were presented in **Table 1**.

**Table 1:** Demographic and clinical characteristics of copd patients stratified by anxiety and depression severity [cases (%), (mean ± SD)].

Group	Asymptomatic group (n=46)	Mild group (n=55)	Moderate-to-severe group (n=14)	χ2/F	*P-value*
Age (years)	67.14 ± 7.56	68.35 ± 6.13	67.56 ± 5.82	0.42	0.661
Gender				3.134	0.209
Woman	21 (45.65)	21 (41.18)	9 (17.65)		
Male	25 (54.35)	34 (53.13)	5 (7.81)		
mMRC Grading	1.58 ± 0.42	1.70 ± 0.49*	1.97 ± 0.51*#	3.82	0.025
Regular physical activity	25 (54.35)	21 (38.18)	3 (21.43)	5.601	0.061
BMI(kg/m2)				0.737	0.947
≤18.4	13 (28.26)	17 (30.91)	3 (21.43)		
18.5~24.9	23 (50.00)	27 (49.09)	7 (50.00)		
≥25	10 (21.74)	11 (20.00)	4 (28.57)		
Smokers	13 (28.26)	20 (36.36)	8 (57.14)	3.926	0.140
Disease duration (years)				7.196	0.027
<10	10 (21.74)	22 (40.00)*	8 (57.14)*#		
≥10	36 (78.26)	33 (60.00)	6 (42.86)		
Presence of Comorbidities	30 (34.78)	29 (47.27)	5 (64.29)	4.151	0.126

*P<0.05 compared with the asymptomatic group; #*P* < 0.05 compared with the mild group.

### 3.2 Comparison of serum gal-3 and Th1/Th2 cytokine levels

Serum concentrations of Gal-3, IL-2, IFN-γ, IL-4, and IL-33 differed significantly among the three cohorts (*P*<0.001). *Post hoc* pairwise analyses indicated a stepwise elevation in these biomarkers correlating with symptom severity: levels were significantly higher in both the mild and moderate-to-severe groups compared to the asymptomatic group (*P*<0.05). Moreover, the moderate-to-severe group exhibited significantly elevated serum levels of all measured markers compared to the mild group (*P*<0.05). These findings were summarized in **Table 2**.

**Table 2:** Comparison of serum Gal-3 and Th1/Th2 cytokine levels stratified by anxiety and depression severity (x¯ ± s).

Group	Number of cases	Gal-3 (ng/mL)	Th1		Th2	
			IL-2 (pg/L)	IFN-γ(ng/L)	IL-4 (ng/L)	IL-33 (pg/mL)
Asymptomatic group	46	21.49 ± 5.24	276.30 ± 32.86	179.95 ± 35.19	54.23 ± 10.80	241.37 ± 20.47
Mild group	55	29.65 ± 8.84*	320.74 ± 45.67*	212.61 ± 41.37*	77.54 ± 12.78*	268.86 ± 34.42*
Moderate-to-severe group	14	35.85 ± 6.38*#	388.39 ± 40.73*#	297.51 ± 37.25*#	90.27 ± 17.12*#	338.58 ± 29.25*#
F-value		26.92	44.25	50.25	63.62	41.87
P-value		<0.001	<0.001	<0.001	<0.001	<0.001

Values are expressed as mean ± standard deviation (x¯ ± s).

Gal-3, Galectin-3; IL, Interleukin; IFN-γ, Interferon-gamma.

*P<0.05 compared with the asymptomatic group; #*P* < 0.05 compared with the mild group.

### 3.3 Correlation between serum biomarkers and anxiety/depression scores

Pearson correlation analysis revealed significant positive correlations between serum levels of Gal-3, IL-2, IFN-γ, IL-4, and IL-33 and scores on both the HADS anxiety scores and the HADS depression scores (*P*<0.05). These data indicated that elevated levels of these inflammatory and immune markers were associated with increasing severity of anxiety and depression symptoms (**Table 3**).

**Table 3:** Pearson correlation analysis between serum biomarkers and anxiety/depression scores.

Biomarkers	HADS anxiety score		HADS depression score	
	r	P- values	r	P- values
Gal-3	0.481	<0.05	0.558	<0.05
IL-2	0.630	<0.05	0.662	<0.05
IFN-γ	0.524	<0.05	0.586	<0.05
IL-4	0.362	<0.05	0.469	<0.05
IL-33	0.617	<0.05	0.631	<0.05

HADS, Hospital Anxiety and Depression Scale; Gal-3, Galectin-3; IL, Interleukin; IFN-γ, Interferon-gamma; *r*, Pearson correlation coefficient.

### 3.4 Logistic regression analysis of anxiety and depression symptoms in patients with COPD

To clarify the associated factors of anxiety and depression symptoms in patients with COPD, with “whether anxiety and depression symptoms occurred” as the binary dependent variable (no anxiety and depression = 0, presence of mild and moderate to severe anxiety and depression = 1), the variables with statistically significant differences in the univariate analysis (MRC classification, disease duration) and serum levels of Gal-3, IL-2, IFN-γ, IL-4, and IL-33 were taken as independent variables. At the same time, potential confounding factors such as age, gender, and comorbidities (hypertension, type 2 diabetes, coronary heart disease) were also included. The collinearity degree among the independent variables was evaluated using the variance inflation factor (VIF) and tolerance. The results showed that the VIF values of all independent variables were all less than 5 (ranging from 1.18 to 3.42), and the tolerance values were all greater than 0.2, indicating the absence of severe multicollinearity.

**Figure 2:**
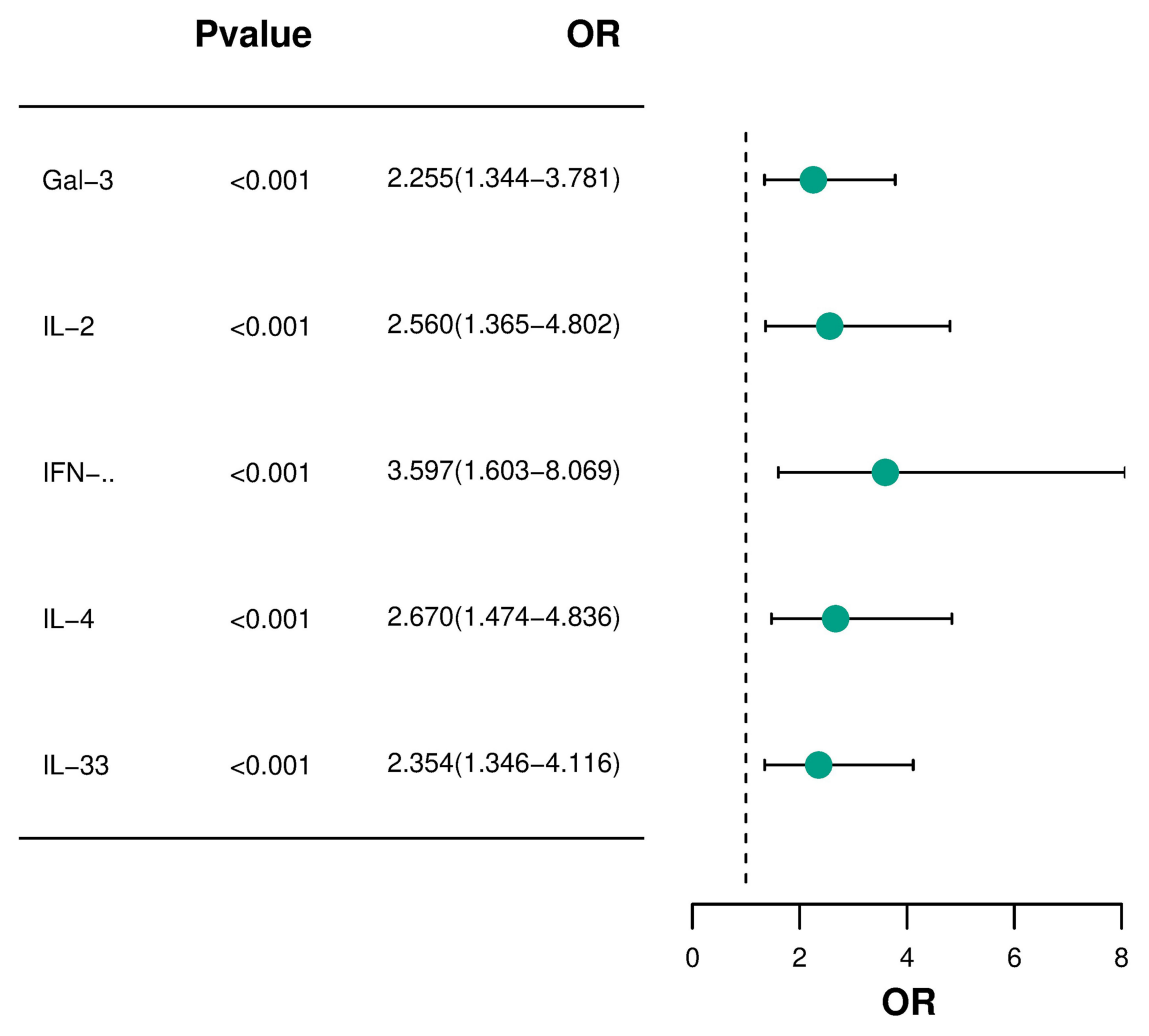
Forest plot of independent risk factors for anxiety and depression in COPD patients.

The results of the multivariate Logistic regression analysis (**Table 4**) showed that, after adjusting for the aforementioned confounding factors, the mMRC classification and disease duration were not associated factors (*P*>0.05), while serum Gal-3, IL-2, IFN-γ, IL-4, and IL-33 were all associated factors for the presence of anxiety and depression symptoms in COPD patients (*P* < 0.05). In the regression model, hypertension (OR = 1.124, 95% CI: 0.756 - 1.672, *P* = 0.562), type 2 diabetes (OR = 1.056, 95% CI: 0.698 - 1.598, *P* = 0.798), and coronary heart disease (OR = 1.201, 95% CI: 0.823 - 1.754, *P* = 0.341) did not show any association with anxiety and depression symptoms. This suggested that these comorbidities did not have a significant confounding effect on the main analysis results of this study. The model was subjected to the Hosmer-Lemeshow goodness-of-fit test, with χ²=5.231 and *P* = 0.732. This indicated that the model fitted well. The VIF values of each independent variable were all less than 5 (Gal-3: 2.14; IL-2: 3.42; IFN-γ: 2.87; IL-4: 2.56; IL-33: 2.91; mMRC grade: 1.18; disease duration: 1.23), indicating the absence of severe multicollinearity. The results were detailed in **Table 4** and **Figure 2**.

**Table 4:** Logistic regression analysis of anxiety and depression symptoms in patients with COPD (associated factors).

Variable	B	SE	Wald	P-value	OR	95%CI
mMRC Grade	0.195	0.188	1.074	0.301	1.215	0.842~1.754
Disease Duration	0.099	0.090	1.208	0.273	1.104	0.927~1.315
Gal-3	0.813	0.264	9.484	<0.001	2.255	1.344~3.781
IL-2	0.940	0.321	8.575	<0.001	2.560	1.365~4.802
IFN-γ	1.280	0.412	9.652	<0.001	3.597	1.603~8.069
IL-4	0.982	0.303	10.504	<0.001	2.670	1.474~4.836
IL-33	0.856	0.285	9.021	<0.001	2.354	1.346~4.116

In the regression model, the comorbidities (hypertension, type 2 diabetes, coronary heart disease) were simultaneously corrected. The OR values and P values for these comorbidities were detailed in Section 2.4 of the results.

*B*, Regression Coefficient; *S.E.*, Standard Error; OR, Odds Ratio; CI, Confidence Interval; mMRC, Modified Medical Research Council Dyspnea Scale; Gal-3, Galectin-3.

### 3.5 ROC curve analysis of serum Gal-3 and Th1/Th2 in diagnosing moderate-to-severe anxiety and depression symptoms in patients with COPD

**Figure 3:**
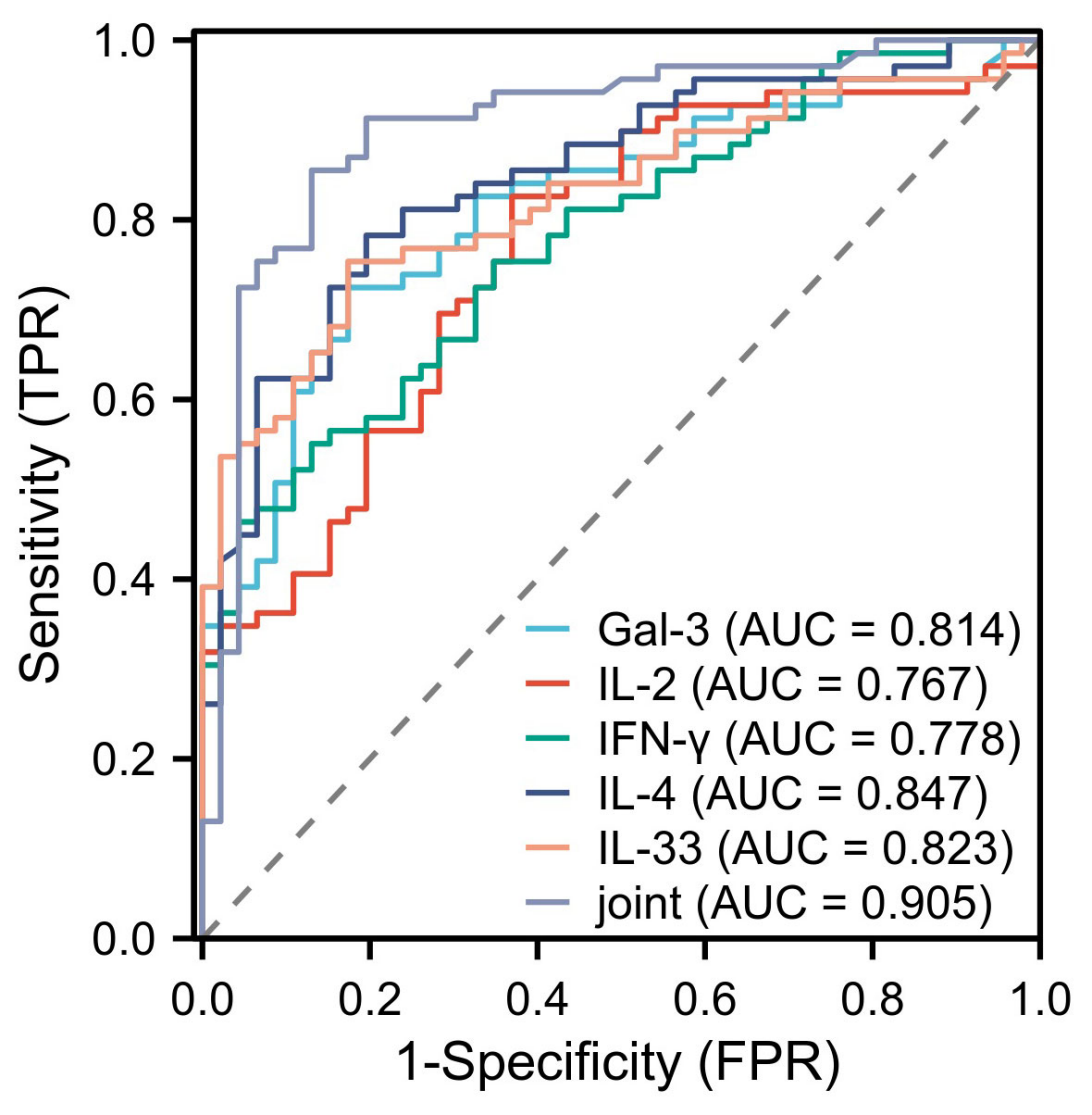
Receiver operating characteristic (ROC) curves of serum Gal-3 and Th1/Th2 cytokines for diagnosing moderate-to-severe anxiety and depression.

ROC curve analysis was utilized to evaluate the diagnostic efficacy of the selected serum markers for identifying moderate-to-severe anxiety and depression. The AUC values for Gal-3, IL-2, IFN-γ, IL-4, and IL-33 were 0.814, 0.767, 0.778, 0.847, and 0.823, respectively. Notably, the combined detection model incorporating all five markers yielded an AUC of 0.905 (95% CI: 0.845–0.965). This combined model demonstrated a sensitivity of 85.51% and a specificity of 86.96%, with a Youden index of 0.725, significantly surpassing any single biomarker. These results were illustrated in **Table 5** and **Figure 3**. Given that the sample size of the moderate-to-severe group was only 14 cases, there might be an optimistic bias in the combined AUC. To conduct internal validation of the combined model using leave-one-out cross-validation, the obtained corrected AUC was 0.872, which was 0.033 lower than the apparent AUC (0.905). This suggested that the discriminative efficacy of the model might decline slightly when applied to new data.

**Table 5:** ROC curve analysis of serum Gal-3 and Th1/Th2 in identifying moderate-to-severe anxiety and depression symptoms in patients with COPD.

Variable	AUC	Sensitivity (%)	Specificity (%)	Youden Index	Cut-off Value	P-value	95%CI
Gal-3	0.814	72.46	82.61	0.551	32.39 ng/mL	<0.05	0.736-0.892
IL-2	0.767	82.61	63.04	0.457	347.26 pg/L	<0.05	0.679-0.854
IFN-γ	0.778	55.07	86.96	0.420	253.94 ng/L	<0.05	0.696-0.861
IL-4	0.847	78.26	80.44	0.587	84.14 ng/L	<0.05	0.776-0.917
IL-33	0.823	75.36	82.61	0.580	297.39 pg/mL	<0.05	0.748-0.898
Combined Model	0.905	85.51	86.96	0.725			0.845-0.965

AUC, Area Under the Curve; CI, Confidence Interval; Gal-3, Galectin-3; IL, Interleukin; IFN-γ, Interferon-gamma; Combined Model included Gal-3, IL-2, IFN-γ, IL-4, and IL-33.

## 3 Discussion

This study provided a systematic analysis of the correlation between serum Gal-3 and Th1/Th2-associated cytokines with the severity of anxiety and depression in patients with COPD. Our findings demonstrated a stepwise elevation in serum Gal-3, IL-2, IFN-γ, IL-4, and IL-33 levels corresponding to the progression of psychiatric symptoms from asymptomatic to moderate-to-severe. Multivariate analysis identified these markers as associated factors for concurrent anxiety and depression in this population. Furthermore, the combined analysis of these biomarkers exhibited certain auxiliary assessment value for identifying moderate-to-severe emotional disorders, providing empirical evidence to support early clinical recognition and intervention strategies.

Galectin-3, a multifunctional inflammatory mediator, appears to act as a critical molecular bridge linking the peripheral pathology of COPD to the onset of anxiety and depression. The hallmark of COPD is chronic airway inflammation, a process that stimulates the systemic release of Gal-3. Crucially, previous studies have suggested that Gal-3 may traverse the blood-brain barrier and activate microglia within the central nervous system (CNS) (17, 18). Microglial activation is a seminal event in neuroinflammation. In theory, it can further induce the release of pro-inflammatory cytokines such as TNF-α and IL-6, which subsequently impair neuronal function and disrupt neurotransmitter homeostasis, ultimately precipitating or exacerbating psychiatric symptoms (18, 19). However, this study did not directly verify the process by which Gal-3 crossed the blood-brain barrier and activated microglia. This mechanism is merely a potential pathway explanation proposed based on the existing literature for the observed results. Previous study has reported elevated serum Gal-3 in patients with major depressive disorder, implicating it in the pathogenesis of depression (20). Our study corroborated these findings within the specific context of COPD, showing that patients with moderate-to-severe anxiety and depression exhibited significantly higher Gal-3 levels compared to those with mild symptoms or no symptoms. This association was further supported by logistic regression analysis (OR = 2.255), aligning with community-based trends reported by King et al. (21). The novelty of the present study lies in establishing this correlation specifically within the chronic inflammatory milieu of COPD. Mechanistically, the systemic inflammatory response in COPD may activate the sympathetic nervous system and the HPA axis, promoting the release of catecholamines and glucocorticoids. This neuroendocrine dysregulation can further amplify inflammatory signaling, creating a vicious cycle that fosters the development of anxiety and depressive states (22, 23). Animal experiments have provided preliminary evidence for the causal role of Gal-3 and inflammatory factors in mood disorders. Stajic et al. (24) found that under basal physiological conditions, Gal-3 knockout mice exhibited significant anxiety-like behaviors, accompanied by a decrease in the expression of brain-derived neurotrophic factor (BDNF) and GABA-A receptor subunits in the hippocampus, suggesting that Gal-3 may have an endogenous anti-anxiety protective effect under normal conditions. However, under the acute neuroinflammatory condition induced by LPS, the absence of Gal-3 actually alleviated the anxiety-like behaviors induced by the inflammation, indicating that Gal-3 can transform into a pro-inflammatory pathogenic factor in a pathological inflammatory environment. The above results reveal the bidirectional regulatory property of Gal-3: its function depends on the inflammatory background. This is in complete agreement with the observation in this study that the elevated level of Gal-3 in the chronic inflammatory state of COPD is accompanied by the aggravation of anxiety and depression symptoms. In a persistent and systemic inflammatory background, the excessive expression of Gal-3 may drive neuroinflammation and promote the occurrence of emotional disorders. Furthermore, Pelgrim et al. (25) demonstrated through a COPD mouse model (induced by elastase for pulmonary emphysema and LPS for pulmonary inflammation) that lung lesions can lead to brain function impairment by disrupting the integrity of the blood-brain barrier. Behavioral tests also revealed changes in cognitive and emotional-related behaviors, further supporting the central role of the lung-brain axis in the brain dysfunction of COPD. Liu et al. (26) demonstrated in a rat model of COPD with comorbid depression that esketamine could alleviate inflammation in the lungs and hippocampus by inhibiting the MAPK/NF-κB signaling pathway, and regulate the composition of the intestinal microbiota and short-chain fatty acid metabolism, thereby improving the depressive-like behaviors and lung function of COPD rats. This provided direct experimental evidence for the pathogenic role of the “gut-lung-brain axis” in the comorbid emotional disorders of COPD. The above studies collectively indicate that Gal-3 is likely to be a key pro-inflammatory mediator in the lung-brain axis, mediating the transmission of peripheral pulmonary inflammation to the central nervous system, thereby participating in the occurrence and development of anxiety and depression symptoms in patients with COPD.

We also observed that the Th1 cytokines IL-2 and IFN-γ were significantly elevated as the anxiety and depression symptoms of COPD patients worsened. Both were positively correlated with the HADS anxiety scores and the HADS depression scores, and were associated factors (IL-2: OR = 2.560; IFN-γ: OR = 3.597). Th1 cytokines are primary mediators of cellular immunity, and their aberrant elevation suggests that immune dysregulation contributes to the pathogenesis of emotional disorders (27). Animal models by Dos Santos et al. (28) have demonstrated that elevated IFN-γ suppresses nitric oxide production in the brain, attenuates the response to antidepressants, and exacerbates anxiety-like behaviors findings that resonate with our clinical data. In COPD, persistent airway inflammation may drive Th1 activation and the secretion of IL-2 and IFN-γ. It is speculated that these cytokines may penetrate the CNS via the peripheral immune-brain axis, stimulating the HPA axis and leading to excessive glucocorticoid secretion, which is known to cause structural damage to mood-regulating regions such as the hippocampus (29, 30). Additionally, IL-2 may directly influence neuronal proliferation and survival, contributing to a neuroinflammatory microenvironment, while IFN-γ may induce neuronal apoptosis and compromise neural circuit integrity, collectively driving the progression of psychiatric symptoms (31). The above-mentioned mechanism pathway is constructed based on indirect evidence from existing literature. Its direct applicability in the COPD patients with anxiety and depression group in this study still requires further verification through basic experiments.

In this study, the levels of Th2-type cytokines IL-4 and IL-33 were significantly elevated in the moderate-to-severe anxiety and depression group, and were positively correlated with the anxiety and depression scores. They were identified as associated factors (IL-4: OR = 2.670; IL-33: OR = 2.354). This result may seem to differ from the anti-inflammatory and neuroprotective effects of IL-4 observed in some studies, but it actually reflects the complexity of the immune-inflammatory network in patients with COPD. IL-4, as a core member of Th2-type cytokines, has been reported in studies that it can protect neuronal functions by regulating the PPARγ activity of microglia (32, 33). However, in this study, its level increased as anxiety and depression worsened, suggesting that it might be related to the “inflammatory compensatory response” in patients with COPD. Under the chronic inflammatory state of COPD, the balance between Th1 and Th2 is disrupted. The increase in IL-4 may reflect the compensatory regulation of the body to counteract the excessive activation of Th1-type inflammation. However, this compensation may not be sufficient to reverse the damage caused by neuroinflammation. It is speculated that it may participate in the occurrence of mood disorders through other pathways (34). Regarding the role of IL-4 in the co-occurring emotional disorders in COPD patients, the current evidence is insufficient and further research is needed to distinguish between protective and pathogenic roles. Corlateanu et al. (35) propose in the systematic review that there exists a “malignant psychological cycle” between COPD and depression. COPD triggers depression through chronic inflammation and hypoxemia, and depression, in turn, exacerbates COPD through decreased treatment compliance and smoking behavior. This review indicates that IL-2 can cross the blood-brain barrier and cause neurocognitive dysfunction, and IFN-γ administration can induce depressive-like symptoms, providing independent evidence to support the finding of elevated Th1-type cytokines in this study. IL-33 acts as a potent alarmin and pro-inflammatory cytokine that may exacerbate both airway and systemic inflammation by activating mast cells and eosinophils (36). Its elevation in severe cases suggests it may amplify neuroinflammatory cascades, thereby worsening anxiety and depression. This speculative mechanism also requires further basic research to be confirmed. A total of 115 COPD patients were included in this study. Among them, only 14 cases (12.17%) had moderate-to-severe anxiety and depression symptoms. This proportion was roughly consistent with the epidemiological reports on moderate-to-severe anxiety and depression in stable COPD patients. According to the general principle in multivariate Logistic regression analysis that each independent variable needs at least 10 to 15 outcome events, in this study, 7 independent variables (MRC classification, disease duration, Gal-3, IL-2, IFN-γ, IL-4, IL-33) were included in the regression model. 14 patients with moderate-to-severe conditions reached the sample size threshold for fitting the model (14/7 = 2 cases per variable), but it was lower than the ideal ratio of events per variable (≥ 10). Therefore, the Logistic regression and ROC analysis conducted in this study were all exploratory analyses, and the results need to be further verified in a prospective study with a larger sample size.

Prior research on Th1/Th2 imbalance in COPD has predominantly focused on acute exacerbations (37) or immune cell differentiation (38), often lacking a systematic evaluation of psychiatric comorbidities in stable patients. Similarly, studies on Gal-3 have largely been confined to its role in lung function decline and prognosis (39, 40), neglecting its potential impact on mental health comorbidities. Compared with conventional subjective scale assessments such as HADS, the detection of serum Gal-3 and Th1/Th2 cytokines has the following advantages. Firstly, the assessment of the scale relies on the patients’ subjective reports of their own emotional states. COPD patients often underestimate or confuse emotional symptoms due to physical symptoms such as breathing difficulties and fatigue. Moreover, factors such as education level and psychological defense mechanisms can affect them, potentially leading to missed diagnoses or misdiagnoses. Research shows that approximately 30% to 50% of patients with COPD who also suffer from depression have not been identified by clinical means (41). Secondly, serum biomarkers, as objective laboratory indicators, are not affected by subjective reporting biases, have good repeatability, and can be completed simultaneously with routine biochemical tests. This makes them useful for implementing “opportunistic screening” of emotional disorders during lung function follow-up or during hospitalization. The increase of these biomarkers reflects the pathological and physiological activation state of the “lung-brain axis”, and holds potential mechanistic implications. The latest review by Yu et al. (42) on the lung-brain axis points out that the oxidative stress and inflammatory response in the lungs of COPD patients can “leak” through the systemic circulation, which damages the integrity of the blood-brain barrier, allows pro-inflammatory factors such as IL-6 and TNF-α to enter the central nervous system, and over-activates microglia and causing dysfunction of the HPA axis. This ultimately leads to neuronal damage in the hippocampus and prefrontal cortex and impairing emotional regulation. In this study, the increase of Gal-3 can reflect the infiltration of peripheral inflammation into the central nervous system, while the imbalance of Th1/Th2 indicates a systemic immune polarization state. The combination of these two factors not only can serve as an auxiliary assessment tool for anxiety and depression related to COPD, but is also likely to reflect different links in the continuous pathological process from peripheral immune activation to central nervous inflammation in the “lung-brain axis”. Among them, the increasing trend of IL-4 in this study is consistent with the Th2 bias observed in simple depression, but it does not indicate a neuroprotective effect. Instead, it represents a compensatory-feedback of the body’s response to the Th1 dominance in the context of chronic inflammation of COPD. However, this compensation is insufficient to reverse the neuroinjury driven by the excessive activation of the lung-brain axis. Similarly, as an alarm factor derived from airway epithelial cells, the increased release of IL-33 directly reflects lung tissue damage and inflammatory signals. This signal can be transmitted through the systemic circulation to the central nervous system, which further amplifies the neuroinflammatory effect, demonstrating the pathway in which pulmonary-derived signals drive brain dysfunction in the “lung-brain axis” (35). Therefore, by combining the conventional clinical scale assessment with serum biomarker detection, the shortcomings of subjective assessment can be compensated, providing the clinical field with an auxiliary assessment method that is both objective, early-sensitive, and mechanism-oriented.

This study, through ROC curve analysis, found that although each individual indicator (Gal-3, IL-2, IFN-γ, IL-4, IL-33) showed certain evaluation value (with an AUC ranging from 0.767 to 0.847), the evaluation efficiency of their combined detection was significantly improved, with an AUC as high as 0.905, and the sensitivity and specificity reaching 85.51% and 86.96% respectively. The efficacy of this combined model is not only superior to any single indicator, but also exceeds the assessment level of individual inflammatory markers reported in some previous studies (11). This study not only included the classic Th1/Th2 cytokines (IL-2, IFN-γ, and IL-4), but also combined IL-33, which is closely related to Th2 immunity and tissue inflammation. It also used the HADS scale to simultaneously assess the anxiety and depression dimensions, covering a more comprehensive spectrum of emotional disorders. The results of this study revealed that in the context of chronic inflammation in COPD, the inflammatory response mediated by Gal-3 and the Th1/Th2 immune imbalance may jointly constitute an important part of the peripheral immune inflammation-central nervous system dysfunction pathway, and play a synergistic role in the occurrence and development of anxiety and depression. In the ROC analysis, the combined detection increased the sensitivity to 85.51%, which was superior to all individual indicators, suggesting that it could reduce false negatives in clinical screening. The specificity was 86.96%, ranking second highest along with IFN-γ, suggesting that the risk of misdiagnosis did not increase due to the combination. However, the combined detection of these five indicators would increase the blood sampling volume and the cost of the ELISA reagent kits in clinical practice. Based on the data from this study, the combined detection method showed an increase of 6.8% in AUC compared to the method of only detecting IL-4, and the accuracy index improved by 0.138. This meant that for every 100 screenings, approximately 5 to 6 cases of moderate-to-severe anxiety and depression can be correctly identified additionally. Whether this net gain can be balanced against the additional testing costs still needs to be verified through further health economics evaluation (cost-effectiveness analysis). It is suggested that future research should, on the basis of expanding the sample size, select the top 2 to 3 core indicators with the greatest contribution through stepwise regression or LASSO algorithm, in order to maintain the diagnostic efficacy while reducing the testing cost. In the ROC analysis, the critical values of each indicator are all single-point estimates based on the principle of the maximum correct index. No internal Bootstrap validation is conducted, and the stability of these values may be affected by the sample size.

Although the baseline data showed no statistically significant differences in age, gender, body mass index, smoking history, and comorbidities among the groups, there were still several COPD disease characteristics that were not included in the analysis and might have confounding effects on the results. The mMRC classification only reflects the subjective perception of dyspnea and fails to cover objective indicators such as the degree of lung function impairment (FEV1%pred), the number of acute exacerbations in the past year, resting blood oxygen saturation, long-term home oxygen therapy history, and the use of systemic glucocorticoids. Repeated acute exacerbations can simultaneously aggravate peripheral inflammatory responses and psychological stress. Persistent hypoxemia directly damages cerebral vascular endothelium and activates the hypothalamic-pituitary-adrenal axis. Moreover, long-term exposure to glucocorticoids is clearly associated with an increased risk of emotional disorders (43). Omitting the correction for these variables implies that the observed association between serum indicators and anxiety-depression symptoms may be partially attributed to the overall severity of COPD rather than the specific biological signals of emotional disorders. Secondly, Gal-3 and Th1/Th2 cytokines are essentially non-specific indicators of inflammation or immune activation. Previous studies have confirmed that Gal-3 is independently associated with the degree of airflow limitation and the risk of acute exacerbation in COPD. IFN-γ and IL-2 reflect the intensity of Th1-type inflammation, while IL-33 indicates airway epithelial damage and type 2 immune activation (39). Therefore, the elevated levels of the above indicators in the patients with moderate-to-severe anxiety and depression may collectively reflect the “severity of the disease-systemic inflammatory load-emotional symptoms” complex state. When used alone for the assessment of mental symptoms, they may lack specificity. Thirdly, since no healthy control group was set up and only patients with anxiety and depression (without COPD) were included, it was impossible to separate the effect of underlying diseases from the effect of emotional symptoms, nor could it be determined whether the observed biological marker profile was a unique pattern in the co-occurring state of COPD and anxiety/depression. Given the aforementioned design limitations, the results of this study can only be interpreted as the association between serum indicators and the severity of emotional symptoms within the stable phase COPD patient population. They should not be extrapolated to independent diagnoses or other clinical scenarios. In addition, social psychological and behavioral factors such as socioeconomic status, years of education, marital status, level of social support, and sleep quality may also simultaneously affect the inflammatory level and the score of the emotional scale. Failure to include these variables may introduce residual confounding.

When analyzing the correlation between serum Gal-3 and Th1/Th2 cytokines and the anxiety and depression symptoms of COPD patients, the severity of the disease and the treatment history are essential potential confounding factors that must be taken into consideration. Previous studies have confirmed that the severity of COPD itself is closely related to the level of inflammation and emotional disorders: for every 10% decrease in FEV1%pred, the risk of depression in patients increases by approximately 15% to 20% (44); Frequent acute exacerbations (≥ 2 times per year) can lead to continuous elevation of inflammatory factors such as IL-6 and TNF-α, while also exacerbating psychological stress (45); Resting hypoxemia (SpO2 ≤ 88%) can directly damage the integrity of the blood-brain barrier, activate microglia cells, and promote neuroinflammation (13). Furthermore, the history of treatment also constitutes an important source of confounding factors: long-term systemic use of glucocorticoids has been proven to induce or exacerbate depressive symptoms, and the mechanism involves impaired negative feedback inhibition of the HPA axis and damage to hippocampal neurons (46); while regular inhalation of glucocorticoids combined with long-acting bronchodilators may indirectly improve emotional state by reducing the overall inflammatory burden (47). Although this study adjusted for mMRC classification (subjective perception of breathing difficulty) and disease duration, it did not include the aforementioned objective indicators (FEV1%pred, frequency of acute exacerbations, resting blood oxygen saturation, history of long-term oxygen therapy, specific medication regimens of glucocorticoids, etc.). Therefore, the observed associations between Gal-3 and Th1/Th2 cytokines and anxiety/depression may be partially attributed to the overall severity of COPD rather than specific biological signals of emotional disorders. Future studies should incorporate these variables as mandatory correction factors into the multi-factor model, or control for confounding bias through methods such as propensity score matching.

## 4 Conclusions

In summary, the levels of serum Gal-3 and Th1/Th2-related cytokines in COPD patients were closely related to the severity of anxiety and depression symptoms, and they were influencing factors. The combined detection of these five indicators had a high auxiliary assessment value for identifying moderate-to-severe anxiety and depression symptoms. These markers were associated with moderate-to-severe symptoms and may be helpful for clinical stratification.

This study has the following limitations: It adopted a single-center cross-sectional design, which precluded the ability to infer causal relationships, and required a prospective cohort study for verification. Those who were not included in the health control group and those with simple anxiety or depression could not rule out the independent influence of the underlying COPD disease on the serum indicators. The number of cases in the moderate-to-severe anxiety and depression group was only 14. The ratio of event count to independent variable was approximately 2. The estimated Logistic regression coefficient may be unstable. In the ROC analysis, the combined AUC may have an optimistic bias. The leave-one-out cross-validation corrected AUC dropped to 0.872. The current combined model was in the preliminary exploration stage. Variables such as the degree of lung function impairment, the frequency of acute exacerbations, hypoxemia, the history of glucocorticoid use, and sleep quality, which were related to the severity of COPD, were not included. This might introduce residual confounding. Serum Gal-3 and Th1/Th2 cytokines were all non-specific indicators of inflammation. Their elevation may collectively reflect the severity of the disease and the systemic inflammatory load. A comprehensive assessment was conducted using the HADS scale. However, the use of multi-dimensional tools such as PHQ-9, GAD-7, or DASS-21 was not employed, which limited the acquisition of information on the independent dimensions of anxiety and depression. No health economics evaluation was conducted for the combined testing scheme. Future research can be further developed in the following directions: establish a mouse model of COPD combined with anxiety and depression-like behaviors, and intervene in Gal-3 or Th1/Th2 pathways to verify the causal mechanism of the lung-brain axis; conduct multi-center prospective cohort studies to verify the external applicability of the combined model; incorporate multi-dimensional emotional assessment tools to clarify the specific associations between biomarkers and the independent dimensions of anxiety and depression. Furthermore, although this study adjusted for common comorbidities such as hypertension, type 2 diabetes, and coronary heart disease in the regression model, due to the limited sample size, no subgroup analysis was conducted based on the types of comorbidities. Thus, the potential differences in the strength of the association between serum indicators and anxiety and depression symptoms under different comorbidity backgrounds could not be evaluated. Future studies can expand the sample size and conduct subgroup analyses to clarify the modifying effects of comorbidities. In addition, the smaller positive group may cause the diagnostic performance of the combined test to appear better than it actually is. The decrease in corrected AUC reflects this bias.

## Data Availability

The original contributions presented in the study are included in the article/supplementary material. Further inquiries can be directed to the corresponding author.
